# Supplementary material for: Polymorphisms in Pfkelch13 domains before and after the introduction of artemisinin-based combination therapy in Southwest Nigeria
Source: PLoS One. 2025 Mar 31;20(3):e0316479. doi: 10.1371/journal.pone.0316479 (PMC11957316; doi:10.1371/journal.pone.0316479)

Samples: 14412
Bases: 833
Average spacing: 18

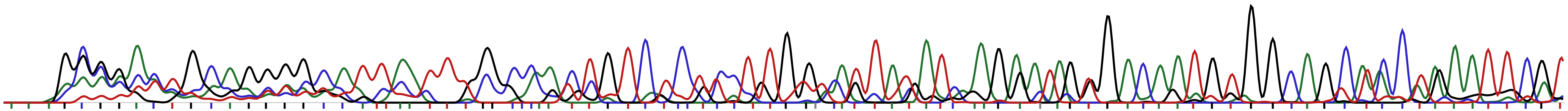
10 20 30 40 50 60 70 80 90
A A A G C G G A C T G C A G G G G C C A TT AA T T T GG CCCAA T T G T C T CC T C C T T G G - CAT T A T A T C AA G A - A G T G A C A A T G T G G C A G C T C C T A A A T T C G T

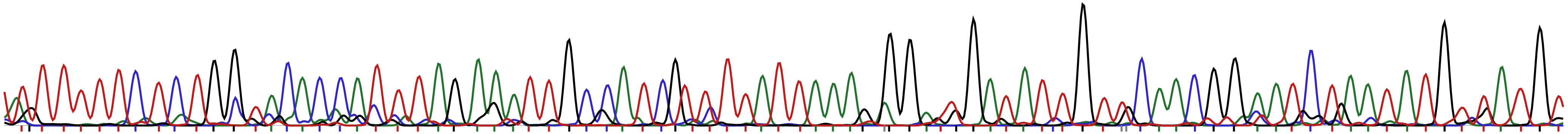
100 110 120 130 140 150 160 170 180
TGT T T T T C T C T G G T A C A C C A T T T A G A A A T T G C C A T C G T T T T A T T A A A G -G G A T G G A T A T C T G T -- C A A C G G A A T C T A A T A T G T T A T G T

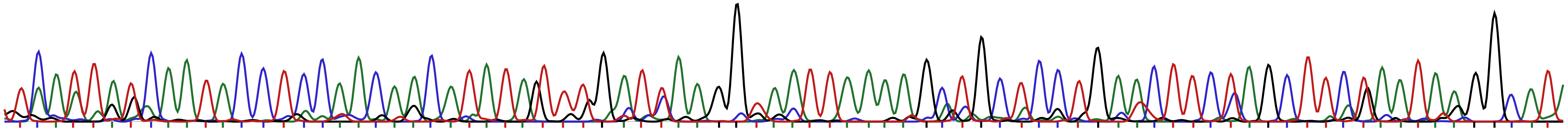
190 200 210 220 230 240 250 260
T C A T T A T C A A T A C C T C C A A C A A C A T A T A T T T G A T T A A G G T A A T T A A A A G C T G C T C C T G A A C T T C T A G C T T C T A A T A A G G C A T

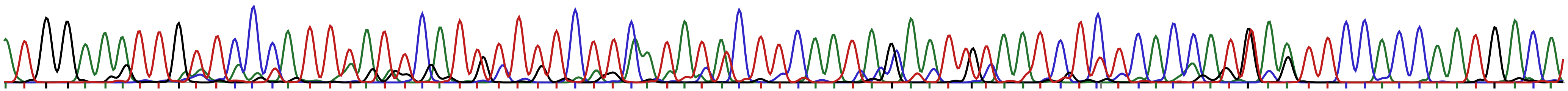
270 280 290 300 310 320 330 340
A T G G A A A T T G T T C C C A T T T A T T C A T T T T T T C T T C A T A T A C T T C A A T A G A A T G T A A T C T C- T C A C C A T G A A T T C C A C C A A T G A C A

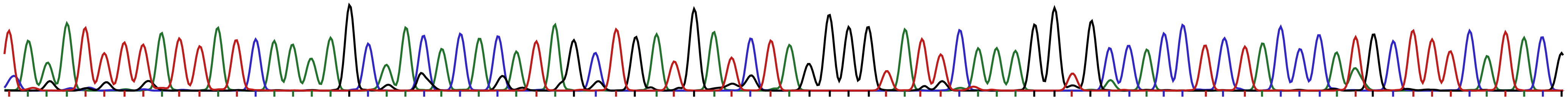
350 360 370 380 390 400 410 420 430
T A A A T T T T A T T A T C A A A A G C A A C A C A C A T A G C T G A T G A T C T A G G G G T A T T C A A A G G T G C C A C C T C T A C C C A T G C T T T C A T A C G

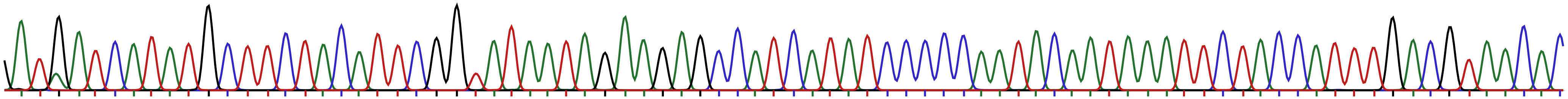
440 450 460 470 480 490 500 510
A T G A T C A T A T G C T T C T A C A T T C G G T A T A A T A G A A G A G C C A T C A T A T C C C C C A A T A C A A T A A A T T C T A C C A T T T G A C G T A A C A C

Samples: 14412
Bases: 833
Average spacing: 18

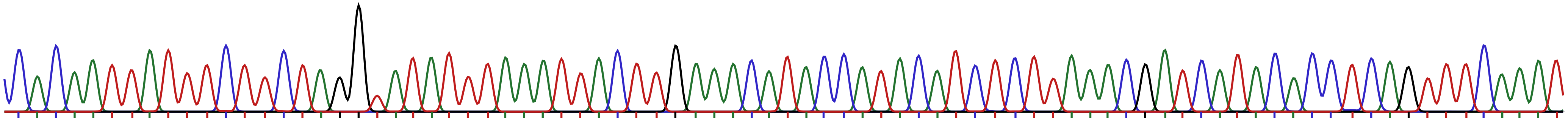

520 530 540 550 560 570 580 590

C A C A A T T A T T T C T T C T A G G T A T A T T T A A A T T A C T T G A A A C A T A C C A T A C A T C T C T T A A A C G A T C A T A C A C C T C A G T T T C A A A T

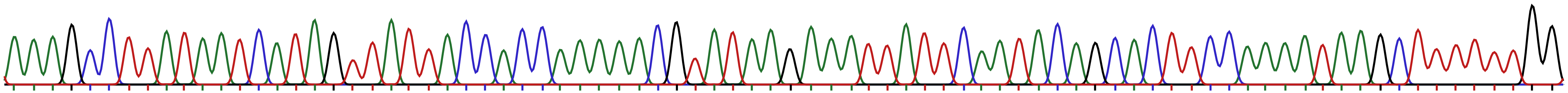

600 610 620 630 640 650 660 670

A A A G C C T T A T A A T C A T A G T T A T T A C C A C C A A A A A C G T A T A A G A A A T T A T T C A A T A C A G C A C T T C C A A A A T A A G C T T T T T T G G

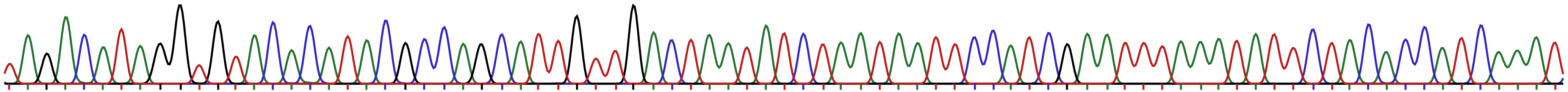

680 690 700 710 720 730 740 750 760

T A G A C A T A G G T G T A C A C A T A C G C C A G C A T T G T T G A C T A A T A T C T A A T A A T T C C A T C G A A T T T A A A T A T T C T A C A C C A T C A A A T

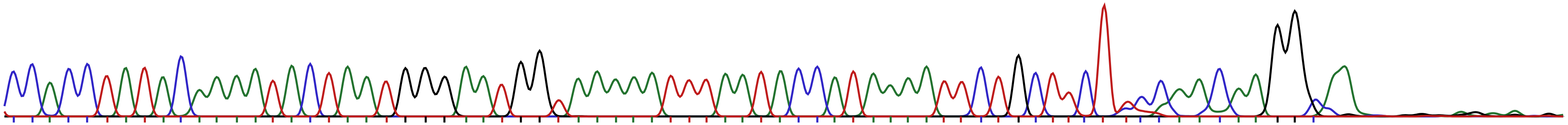

770 780 790 800 810 820 830

C C A C C T A T A C A A A A T A C T A A T G G G A A T G G T A A A A A T T T A A T A C C A T A A A A T T C T G C T T C T T C C A A C A A G G C

Samples:	14412
Bases:	833
Average spacing:	18

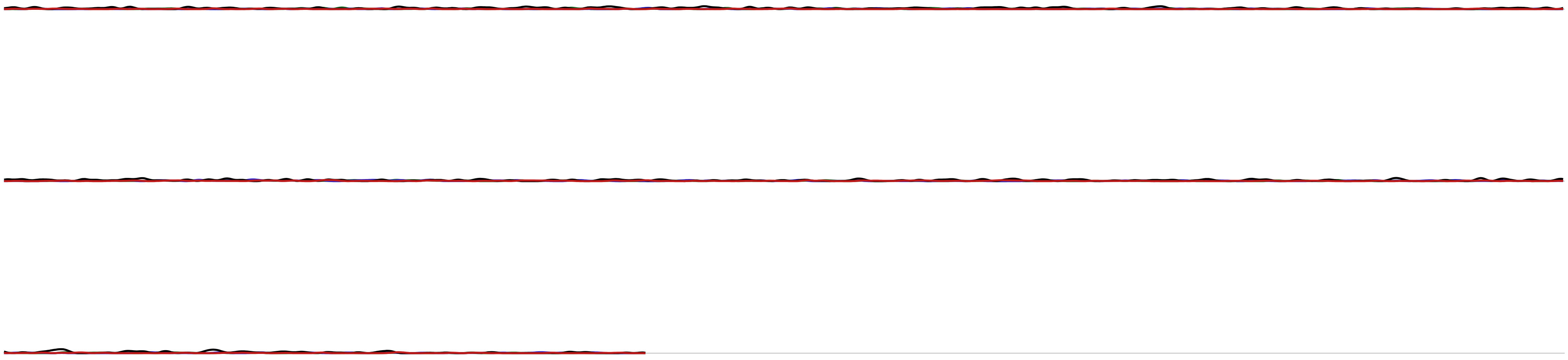

Supplement: Supporting information 2 — (ZIP) [file pone.0316479.s002.zip › 050KN1R_PREMIX_Plate_KELCH1_D03.pdf]
